# Exclusion and Trapping Mechanisms of Boron in Forage Grasses Irrigated with Treated Oilfield-Produced Water

**DOI:** 10.3390/plants15111613

**Published:** 2026-05-24

**Authors:** Khaled Al-Jabri, Mushtaque Ahmed, Ahmed Al-Busaidi, Mansour Al-Haddabi, Rhonda R. Janke, Alexandros Stefanakis

**Affiliations:** 1 Department of Soils, Water and Agricultural Engineering, Sultan Qaboos University, Al-Khoud 123, Oman; ahmed99@squ.edu.om (A.A.-B.); mans99@squ.edu.om (M.A.-H.); 2 Department of Plant Sciences, Sultan Qaboos University, Al-Khud 123, Oman; rrjanke@ksu.edu; 3 Laboratory of Environmental Engineering and Management, School of Chemical and Environmental Engineering, Technical University of Crete, 73100 Chania, Greece; astefanakis@tuc.gr

**Keywords:** boron, oilfield-produced water, biochar, soil profile, nature-based solutions, irrigation, salinity, translocation and bioaccumulation factors

## Abstract

The reuse of treated oilfield-produced water (PW) presents a viable solution to water scarcity in arid regions; however, elevated boron (B) levels pose a significant constraint for sustainable irrigation. This study evaluates boron dynamics in a soil–plant system irrigated with treated PW and examines the effectiveness of nature-based solutions in mitigating its accumulation. A controlled experiment using two soil types and multiple water sources was conducted, with biochar and gypsum applied as soil amendments. Boron concentrations were assessed in plant tissues, roots, and soil layers. Results showed significant boron accumulation under PW irrigation, exceeding safe agronomic thresholds, and soil analysis indicated greater boron retention in surface layers. Boron concentrations reached maximum average concentrations exceeding 200 mg kg^−1^. To elucidate species-specific tolerance mechanisms, bioaccumulation factors (BAFs) and translocation factors (TFs) were calculated. Results revealed a distinct root-trapping strategy, with high BAF values under oilfield-produced water, while TF values remained significantly lower, indicating that these forage species successfully restricted boron translocation to aerial tissues.

## 1. Introduction

Oilfield-produced water (PW) from oil and gas processing is one of the industrial sources of treated petrochemical wastewater in Oman [1]. The daily amount of PW accompanied by oil is expected to increase in some of the oil fields and can reach more than 10 times that of produced hydrocarbons [2].

Oilfield-produced water quality is challenging due to its solids content, nutrients concentrations, organic load, hydrocarbon pollutants, salinity, heavy elements, pH value, toxic compounds, and emerging pollutants. PW from oilfields is considered one of the most challenging areas as inorganic and toxic contaminants are present [3,4].

Agriculture production has faced challenges in sustaining crop productivity due to soil and water salinity. Plants face numerous abnormal, physio-morphological, and biochemical changes under salinity stress, which cause delayed germination, high seedling mortality, poor crop stand, stunted growth, and reduced yields [5]. Soil salinity stresses plants in two ways. Roots face challenges with high salinity soil to extract water, and high concentrations of salts and boron in the plant can also be toxic. Irrigation with saline water accumulated around the external roots has a direct effect on cell growth and associated metabolism; however toxic salts may take time to accumulate within plant tissues before significantly impairing plant function [6]. Boron (B) toxicity constrains crop productivity and quality across various global agricultural regions, often occurring naturally in alkaline, saline soils with limited rainfall and leaching capacity [7]. According to [8], the maximum allowable levels of B in irrigation water typically range between 1 and 2 mg L^−1^ for semi-tolerant plant species like maize.

Boron tolerance varies substantially among crop species, and in some cases even among genotypes within the same species. This makes crop selection a critical consideration when alternative irrigation waters are used. The issue is particularly important in forage systems, where the aim is not merely to sustain plant survival, but to produce harvestable biomass that remains agronomically useful and safe for feed. Available crop-tolerance data indicates that moderately tolerant crops, such as barley and maize, can withstand lower boron concentrations in irrigation water compared to highly tolerant crops such as *alfalfa*, as documented by [9]. Reported threshold values are approximately 3.4 mg L^−1^ for barley, 2.0–4.0 mg L^−1^ for maize, 4.0–6.0 mg L^−1^ for *alfalfa*, and about 7.4 mg L^−1^ for sorghum [9]. These differences are agronomically important because a water source that is marginally acceptable for one forage species may still cause excessive boron accumulation in another. The problem is further complicated by the fact that boron has a very narrow range between deficiency and toxicity. Plant response depends not only on its concentration in the irrigation water, but also on uptake rate, internal distribution, and the capacity of the plant to limit movement toward sensitive aerial tissues [10]. For this reason, evaluating boron-rich irrigation water requires more than a general assessment of plant growth. It also requires determining whether specific forage species can maintain comparatively low boron concentrations in shoots through exclusion, sequestration, or restricted xylem transport.

Boron stress represents a significant global concern, adversely impacting maize development, yield, and quality, as noted by [11]. The deleterious effects of boron toxicity are rooted in its disruption of metabolic processes involving ribose components essential for adenosine triphosphate (ATP), nicotinamide adenine dinucleotide (NADH), and nicotinamide adenine dinucleotide phosphate (NADPH), primarily attributed to cell wall damage in plants as noted by [12]. This disruption extends to the interference with RNA, free glucose, or ribose, particularly affecting dividing and developing cells. Additionally, excessive boron accumulation in plant leaves disrupts osmotic balance, impacting transpiration flow [13], with documented adverse consequences on agriculture and ecosystems [14].

Assessing total boron accumulation in plant tissues alone is insufficient to understand species-specific tolerance; rather, the translocation factor (TF)—the ratio of boron in aboveground biomass to roots—serves as a critical indicator of a plant’s internal boron mobility and exclusion mechanisms. A high TF indicates efficient boron transport to aerial tissues via the transpiration stream, increasing phytotoxicity risk, whereas a lower TF may suggest root-level exclusion or restricted xylem loading, representing a tolerance strategy. Despite the extensive literature on the negative impacts of excess boron, there remains a limited understanding regarding the effects and mechanisms of various mitigation strategies, especially concerning the use of oilfield-produced water. Thus, the primary objective of this study is to investigate the impact of boron concentration on selected feeding grass plant leaves, roots, soil, and overall plant growth, aiming to alleviate the detrimental effects of boron toxicity. This study provides a targeted analysis of boron dynamics, which was not comprehensively evaluated in our previous work.

This issue is especially relevant in arid and semi-arid reuse systems, where boron commonly occurs together with salinity, sodicity, and limited natural leaching. In Oman, treated oilfield-produced water represents a potentially valuable non-conventional water source, but its reuse remains constrained by dissolved salts and specific ions that may accumulate progressively in the soil–plant system. Recent work from Oman has shown that irrigation with treated oilfield-produced water can increase boron and other ion concentrations in both soils and plant tissues, indicating that ion-specific risks remain even after treatment substantially reduces oil and suspended solids [15]. Similar concerns have been reported in other dryland reuse settings. In Northeast Tunisia, for example, long-term irrigation with treated wastewater increased boron not only in the soil, but also in drainage water and groundwater, highlighting that boron management in reuse systems must be assessed across the broader soil–water continuum rather than only at the plant level [16]. Taken together, these findings identify boron as a key control parameter in water reuse planning for arid agriculture. They also point to a clear knowledge gap: although forage grasses are often proposed for the productive use of marginal waters, boron-focused studies directly comparing forage species in terms of uptake, retention, and translocation under treated oilfield-produced water remain scarce.

The treatment system effectively reduces suspended solids and hydrocarbons, although evaporation across the wetland increases total dissolved solids (TDS), as summarized in [17,18]. The treated produced water effluent used in this study contained a TDS of approximately 12,000 mg L^−1^ and a boron concentration of 5.6 mg L^−1^.

While our previous study [15] established the broad multi-element accumulation profiles under PW irrigation, the current study provides a targeted, deep-dive assessment exclusively focused on boron concentrations, which was beyond the scope of the initial broad-scale water reuse assessment.

The regulatory context further underscores the importance of boron in irrigation water assessment. In arid regions, where treated wastewater and other alternative waters are increasingly reused, boron is often regulated because general salinity indicators alone do not adequately capture crop-specific toxicity risk. Abu Dhabi’s recycled water regulations specify a boron limit of 3 mg L^−1^, whereas the Saudi executive regulations prescribe 0.75 mg L^−1^ for tertiary-treated sewage water and 2.0 mg L^−1^ for dual-treated sewage water intended for reuse [19,20]. Although these regional regulations were developed for recycled water or treated sewage water rather than oilfield-produced water, they remain useful as comparative benchmarks showing that boron is widely recognized as a critical parameter in irrigation water reuse. Omani wastewater reuse regulations similarly identify boron as a regulated parameter for reuse and discharge [21]. These benchmarks are relevant to the present study, as boron concentrations associated with the oilfield-produced water can exceed thresholds in several regional water reuse frameworks. Thus, the practical question is not only whether forage grasses can survive under produced-water irrigation, but whether appropriate crop selection and soil management can reduce boron transfer to shoots and limit its accumulation in the root zone over time. Regulatory acceptability and agronomic performance should therefore be considered together when assessing the reuse potential of treated oilfield-produced water.

This study aims to (i) assess boron accumulation in plant tissues, roots, and soil under treated PW irrigation, (ii) examine the vertical distribution of boron within the soil profile, and (iii) evaluate the effectiveness of biochar as a nature-based solution to mitigate boron toxicity. By providing a focused analysis of boron behavior, this work contributes to refining sustainable irrigation strategies and supports the safe reuse of alternative water resources in arid agricultural systems.

## 2. Results

### 2.1. Boron Accumulation in Plant Tissues

**Figure 1:**
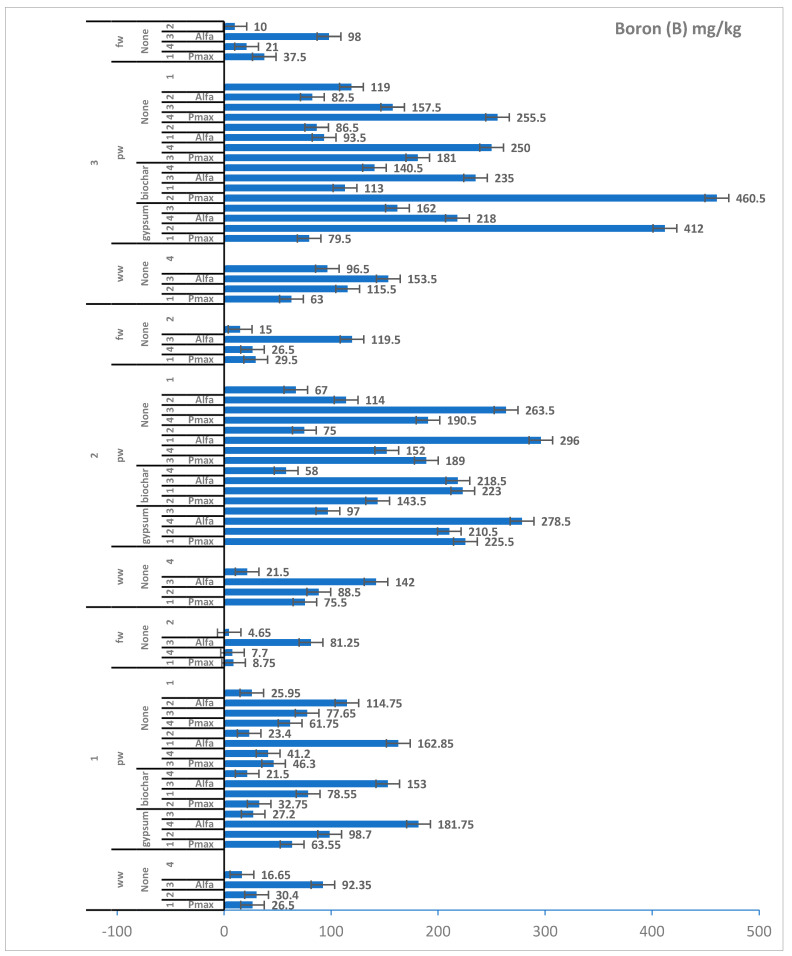
Combined effects of irrigation water levels and treatment conditions on boron content in plant tissues. 1: Harvest one; 2: Harvest two; 3: Harvest three. ww: wastewater, pw: oilfield-produced water, fw: farm groundwater, Pmax: *Panicum maximum*, and Alfa: *Alfalfa*.

**Figure 2:**
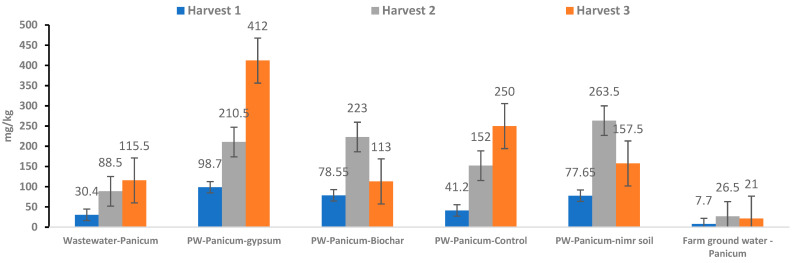
Boron levels in *Panicum* plant tissues of three harvests under different water irrigation types and different soil amendments. PW: oilfield-produced water.

**Figure 3:**
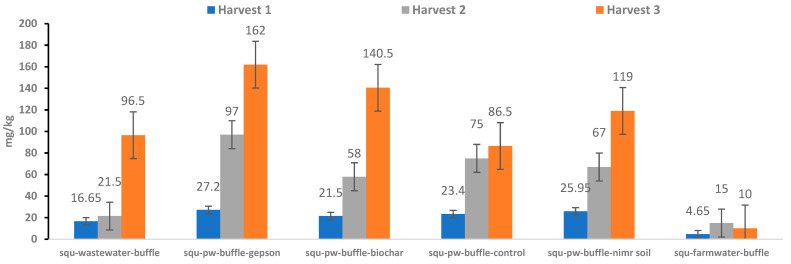
Boron levels in *Buffelgrass* tissues of three harvests under different water irrigation types and different soil amendments. pw: oilfield-produced water.

**Figure 4:**
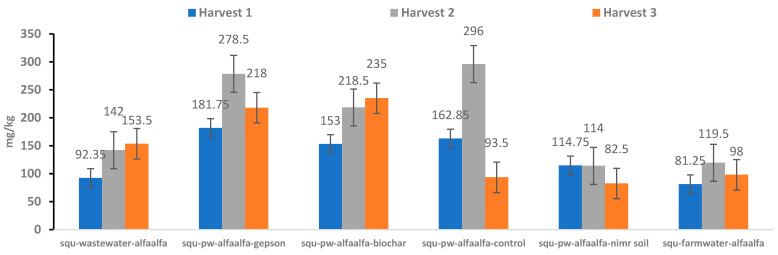
Boron levels in *Alfalfa* tissues of three harvests under different water irrigation types and different soil amendments. pw: oilfield-produced water.

Boron concentrations in plant tissues increased progressively across harvest stages under irrigation with treated oilfield-produced water (PW) (Figure 1, Figure 2, Figure 3 and Figure 4). Statistical analysis (ANOVA) confirmed that this temporal accumulation was statistically significant (*p* < 0.001), with concentrations in several PW treatments reaching maximum averages exceeding 200 mg kg^−1^ by the final harvest, thereby exceeding commonly accepted safe agronomic thresholds. This trend confirms continuous boron uptake and accumulation over time.

Overall, the data confirm that boron dynamics in soil–plant systems are influenced by multiple interacting factors, necessitating a combined analysis of water type, species, and harvest stage when using treated oilfield-produced water for irrigation.

All results are expressed for a saturated extract as the standard soil–water relation for salinity measurements and chemical symmetry [22]. The experiments measured and calculated boron levels in three water types and soils before treatments are outlined sequentially in Table 1 and Table 2.

To evaluate species-specific temporal dynamics, Figure 2, Figure 3 and Figure 4 delineate the boron concentrations in the tissues of *Panicum*, *Buffelgrass*, and *Alfalfa* across the three harvest stages under varying irrigation and amendment regimes. A consistent temporal accumulation pattern was observed across all three forage species, with boron levels progressively increasing from Harvest 1 to Harvest 3. However, the magnitude of this accumulation was distinctly modulated by water quality and species physiology. Under farm groundwater and treated wastewater, boron levels remained relatively low and stable across the harvests. In stark contrast, irrigation with treated produced water triggered a pronounced accumulation curve. Notably, *Alfalfa* exhibited the most aggressive accumulation trajectory under PW, reaching peak concentrations that significantly exceeded the 200 mg kg^−1^ by the third harvest. Conversely, *Buffelgrass* demonstrated a comparatively muted accumulation profile under identical PW conditions, visually reinforcing the root-exclusion mechanism identified in the subsequent translocation factor (TF) analysis. Furthermore, the detailed breakdown by harvest stage confirms that neither biochar nor gypsum effectively arrested this temporal boron accumulation under PW stress, as all amended PW treatments followed the same steep upward trajectory as the unamended controls by the final harvest.

### 2.2. Boron Uptake in Plant Roots

**Figure 5:**
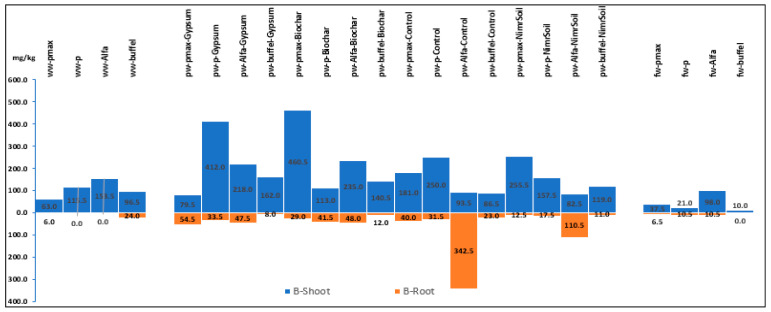
Boron concentration in plant shoots and roots; ww: wastewater, pw: oilfield-produced water, fw: farm groundwater, pmax: *Panicum maximum*, p: *Panicum* and Alfa: *Alfalfa*.

Root analysis confirmed active boron uptake from the soil, with maximum concentrations reaching 342.5 mg kg^−1^ under PW irrigation (Figure 5). Boron levels in roots were consistently lower than in shoots, indicating translocation from roots to aerial plant parts.

Statistical analysis (ANOVA) revealed significant differences in boron accumulation among plant species, with *alfalfa* roots exhibiting the highest concentrations. Soil type also played a critical role, as higher boron accumulation was observed in plants grown in saline Nimr soil compared to control soil. This suggests that soil properties influence boron availability and plant uptake dynamics.

### 2.3. Boron Distribution in Soil Profiles

Boron accumulation in the soil exhibited clear vertical stratification. Soil samples collected at depths of 0–15 cm and 15–30 cm revealed higher concentrations in the upper layer, indicating limited downward mobility and surface accumulation under repeated PW irrigation.

**Figure 6:**
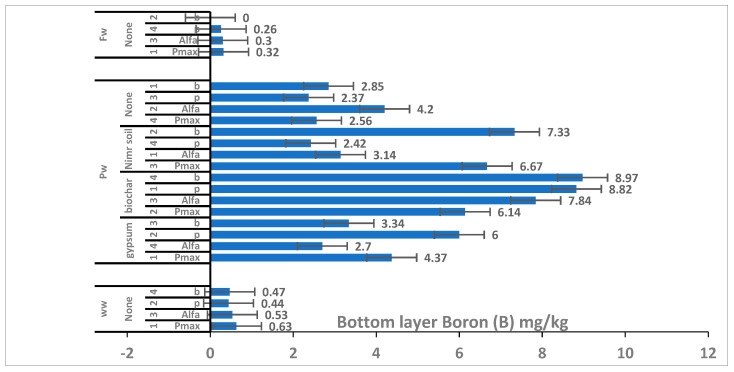
Overall effect of irrigation water and treatment factors on boron concentration in plant bottom soil layers. 1: Replicate 1, 2: Replicate 2, 3: Replicate 3, 4: Replicate 4, ww: wastewater, Pw: oilfield-produced water, Fw: farm groundwater, Pmax: *Panicum maximum*, p: *Panicum*, b: *Buffelgrass*, and Alfa: *Alfalfa*.

The highest boron concentrations recorded were 8.96 mg kg^−1^ in the top layer and 8.97 mg kg^−1^ in the bottom layer under PW treatment (Figure 6). Despite similar peak values, the general trend showed greater accumulation near the surface, highlighting the potential for long-term buildup in upper soil horizons.

### 2.4. Effect of Soil Amendments on Boron Behavior

**Figure 7:**
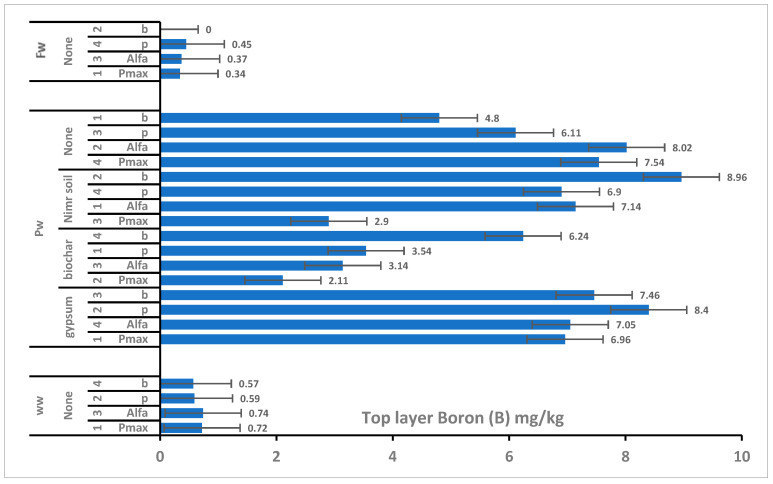
Overall effect of irrigation water and treatment factors on boron concentration in plant top soil layers. 1: Replicate 1, 2: Replicate 2, 3: Replicate 3, 4: Replicate 4, ww: wastewater, Pw: oilfield-produced water, Fw: farm groundwater, Pmax: *Panicum maximum*, p: *Panicum*, b: *Buffelgrass*, and Alfa: *Alfalfa*.

Although biochar is often theorized to influence boron bioavailability through adsorption, our results showed it had limited influence on boron mobility under these specific experimental conditions. The lack of significant reduction in our study suggests that higher application rates or fundamentally different biochar types may be required to achieve boron immobilization in high-salinity produced water systems. In contrast, gypsum showed limited influence on boron mobility, with no significant reduction observed in plant uptake or soil concentration. The highest boron concentrations were consistently observed in aboveground tissues, indicating effective translocation through the transpiration stream. A clear relationship was observed between boron levels in irrigation water (6.95 mg L^−1^ in PW) (Table 2) and accumulation in plant tissues, although uptake was also influenced by plant growth stage and soil characteristics (Figure 1 and Figure 7).

Significant variation was detected among plant species. *Alfalfa* exhibited the highest boron accumulation, while *buffelgrass* showed comparatively lower concentrations, reflecting species-specific differences in boron uptake and tolerance mechanisms.

### 2.5. Interaction Effects

**Figure 8:**
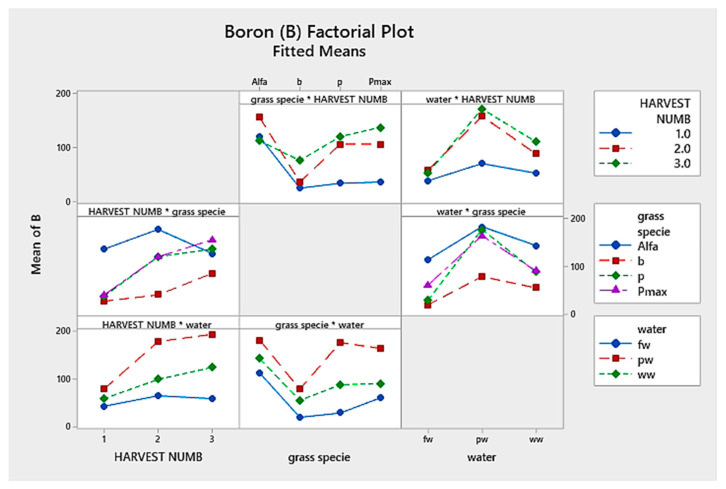
Factorial interaction plots of ANOVA fitted means for boron concentration (mg kg^−1^), illustrating the statistical interactions between (**Top-Left**) grass species and harvest stage, (**Top-Right**) irrigation water type and harvest stage, and (**Bottom-Left**) grass species and water type, and (**Bottom-Right**) the three-way interaction of species, water, and harvest stage. (Abbreviations: fw = farm groundwater; pw = produced water; ww = wastewater; Alfa = *Alfalfa*; b = *Buffelgrass*; p = *Panicum*; Pmax = *Panicum maximum*).

To statistically deconstruct the interacting variables driving boron accumulation, factorial plots of the ANOVA fitted means were generated (Figure 8). The software Minitab 19.1 was used for data organization and calculations. These illustrate the predicted mean boron concentrations calculated by the three-way ANOVA model, holding other factors constant to isolate specific interactions. The top-left panel (grass species × Harvest Number) reveals a strong species-dependent temporal trajectory; while all species accumulated boron over time, the fitted mean line for *Alfalfa* diverged significantly upward by Harvest 3 compared to *Buffelgrass*, which maintained a relatively flat trajectory. The top-right panel (water × Harvest Number) clearly isolates the irrigation effect, demonstrating that the fitted mean for produced water (PW) increased drastically across harvests, while groundwater (fw) and wastewater (ww) remained statistically parallel and low. The bottom-left panel (grass species × water) simplifies this by averaging across harvests, confirming that under PW conditions, *Alfalfa* had the highest predicted mean accumulation, whereas *Buffelgrass* maintained the lowest. Finally, the bottom-right panel integrates all three variables (grass species × water × Harvest Number), which is the core statistical evidence of this study: it visually proves that the significant three-way interaction is driven almost entirely by the extreme upward shift in the PW–*Alfalfa* line over time, while the PW–*Buffelgrass* line remains suppressed, mathematically confirming the species-specific exclusion mechanism under stress.

### 2.6. Boron Translocation Dynamics

**Figure 9:**
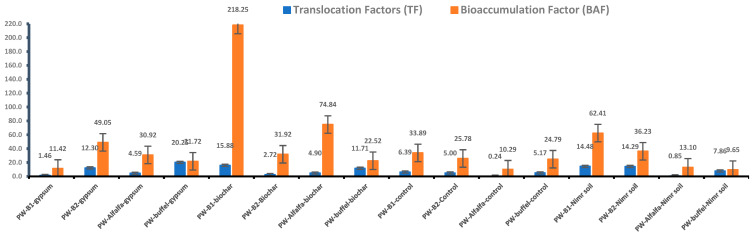
Bioaccumulation and translocation factor of harvest and grass species on boron concentration. Abbreviations: PW = oilfield-produced water; B1 = *Panicum maximum*; B2 = *Panicum*; bufel= *Buffelgrass*.

To quantify the internal partitioning of boron (B) and assess plant tolerance mechanisms, the bioaccumulation factor (BAF; root B/soil B) and translocation factor (TF; shoot B/root B) were calculated for treatments irrigated with oilfield-produced water (PW) (Figure 9).

In contrast, translocation factors (TFs) exposed distinct physiological strategies among the species. *Panicum* species displayed the highest tendency to translocate boron to aerial tissues, with TF values reaching up to 49.05 and 20.25 in certain treatments. *Alfalfa* exhibited an intermediate translocation response, with TF values generally ranging between 11.42 and 14.48. Conversely, *Buffelgrass* exhibited a strict root-exclusion strategy, maintaining drastically lower TF values compared to the other species (ranging from 0.24 to 9.65). Most notably, under specific PW irrigation conditions, *Buffelgrass* achieved a TF of just 0.24, demonstrating near-complete restriction of boron movement from the root system to the consumable aboveground biomass.

## 3. Discussion

The most critical finding of this study is the quantification of species-specific boron-handling behavior among forage species under treated oilfield-produced water (PW) irrigation, revealed through bioaccumulation factors (BAFs) and translocation factors (TFs). While our previous broad-scale assessment [15] highlighted that *Alfalfa* accumulated higher absolute boron concentrations in tissues than *Buffelgrass*, the current study provides a quantitative explanation for this behavior: *Alfalfa* functions as a hyper-translocator, whereas *Buffelgrass* acts as an excluder. This distinction is crucial for evaluating the safe reuse of marginal irrigation waters in arid regions.

The significant differences in boron accumulation observed across the treatments can be directly attributed to the distinct geochemical profiles of the irrigation water sources. Farm groundwater (FW) contained a baseline boron concentration of 0.23 mg L^−1^, while treated wastewater (WW) contained 1.17 mg L^−1^. In contrast, the treated PW used in this experiment contained 6.95 mg L^−1^ B—placing it at or above the upper tolerance range reported for many forage crops and close to the upper end of the range reported for *Alfalfa*-type tolerance [9]. Because boron has a very narrow range between deficiency and toxicity in plants [10], the extreme boron load in PW acted as a severe physiological stressor that exposed the underlying genetic transport differences between the species. Under FW and WW irrigation, boron uptake remained within normal physiological limits, and translocation patterns were relatively uniform. However, under PW conditions, plant response was shaped less by exposure alone than by how boron was handled internally. The present results strongly support that the suitability of a forage species for boron-rich irrigation water cannot be inferred from broad crop-tolerance rankings alone; rather, it depends on whether the species functions as a translocator or an excluder under the actual water quality conditions of the system.

A primary objective of this study was to evaluate the efficacy of nature-based soil amendments to mitigate boron toxicity. Unexpectedly, both biochar and gypsum did not reduce boron uptake under the tested conditions, and biochar was associated with increased root bioaccumulation (BAF up to 218.25). This paradoxical result can be explained by the specific biogeochemistry of boron in high-salinity systems. Boron primarily exists in soil solution as uncharged boric acid [B(OH)_3_], which is highly mobile and does not bind strongly to soil particles. Unlike heavy metals (which are cationic and readily adsorb to biochar surfaces), neutral boric acid does not readily immobilize on biochar. Furthermore, under the extreme salinity conditions of the PW (TDS 6500–7000 mg/L), competitive ion interactions may have altered the biochar’s surface chemistry, potentially rendering its adsorption sites ineffective for boron while simultaneously altering the soil’s osmotic potential. This may have altered plant water uptake dynamics and osmotic conditions, potentially contributing to increased boron uptake into the root zone (explaining the spiked BAF). Mechanistic studies in cereals have shown that true tolerance to elevated boron is usually associated with reduced uptake and enhanced efflux from sensitive tissues [23]; however, neither biochar nor gypsum facilitated this natural efflux mechanism in our high-salinity environment. Similarly, gypsum (CaSO_4_) showed limited influence on boron mobility. While gypsum is highly effective at displacing sodium and reducing sodicity, calcium does not effectively complex with or immobilize uncharged boric acid. Therefore, modifying the soil environment with these specific amendments was insufficient to protect the plants under the unique chemical stress of PW irrigation.

While absolute yield reductions were not quantified in this targeted ion-uptake study, observed shoot concentrations exceeding 200 mg/kg approach or exceed commonly reported phytotoxic thresholds in the literature; however, actual phytotoxic effects could not be confirmed in the absence of biomass or physiological measurements. Although acute physiological phytotoxicity is traditionally confirmed through measurable reductions in biomass or leaf chlorosis, the high magnitude of tissue accumulation strongly suggests that sustained PW irrigation would likely lead to agronomic yield penalties and potentially unsafe fodder quality. Future studies integrating these BAF/TF metrics with long-term biomass tracking are essential to definitively confirm the phytotoxic tipping points for these specific species under arid PW irrigation.

It is also important to acknowledge the inherent variability in calculating ratio-based indices (TF and BAF) from discrete, destructive harvests rather than continuous isotopic tracing. Despite variability in TF and BAF values, consistent directional trends were observed across species and treatments. This consistency supports the interpretation of species-specific boron partitioning behavior under the tested conditions. *Alfalfa* frequently demonstrated TF values greater than 1 across PW treatments, indicating a physiological tendency to translocate a significant proportion of absorbed boron to aerial tissues via the transpiration stream, where it can disrupt metabolic processes and cause cell wall damage [12]. Conversely, *Buffelgrass* consistently maintained TF values well below 1, indicating a physiological restriction at the root-to-shoot boundary. Recent reviews emphasize that such differences in homeostasis whether through exclusion or controlled redistribution largely determine toxicity outcomes [9]. The restriction observed in *Buffelgrass* aligns with the literature suggesting root-level compartmentation strategies to prevent vascular loading [8]. If tolerance relies heavily on internal transport behavior rather than bulk soil concentration alone, amendment-based mitigation alone is likely insufficient as a standalone management strategy.

Overall, these findings demonstrate that the safe reuse of treated oilfield-produced water for forage production in arid regions should not rely solely on chemical soil amendments. Instead, greater emphasis should be placed on crop selection—particularly species demonstrating low translocation trends such as *Buffelgrass*—alongside routine monitoring of soil boron and plant tissue concentrations. This integrated approach offers a more reliable pathway for managing boron risks under PW irrigation.

## 4. Materials and Methods

### 4.1. Experimental Design and Soil Characterization

A factorial pot experiment was conducted at the Agricultural Experimental Station (AES) of Sultan Qaboos University (SQU), Muscat, Oman. The experiment employed a completely randomized design with three independent factors: (i) irrigation water source (oilfield-produced water [PW], treated wastewater [WW], and farm groundwater [FW]); (ii) soil substrate (Nimr saline soil and standard SQU control soil); and (iii) soil amendments (biochar and gypsum). Table 3 provides a summary of third-party water quality analysis for the oilfield-produced water, comparing upstream and downstream samples. The data indicate that while hydrocarbons and suspended solids are effectively removed, evaporation across the wetland increases total dissolved solids (TDS) from 6500–7000 mg L^−1^ at the inflow to 12,000 mg L^−1^ at the outflow, with the final treated outflow containing 5.6 mg L^−1^ of boron.

The Nimr soil was collected from the Oman Nir field area, representing typical arid-zone oilfield soil, while the control soil was obtained from the SQU experimental farm. Prior to the experiment, baseline physicochemical properties of both soils were analyzed, including texture, pH, Electrical Conductivity (EC), and initial boron concentration (Table 1). The soils were air-dried, sieved through a 2 mm mesh, and uniformly packed into 15 L plastic pots.

### 4.2. Soil Amendment Application

Two commonly utilized soil amendments—biochar and agricultural gypsum—were applied prior to planting to test their efficacy in mitigating boron uptake under high-salinity conditions. Biochar, derived from reeds pyrolyzed at a temperature of 500 °C, was applied at a rate of 10 g/kg of soil. Agricultural gypsum (CaSO_4_·2H_2_O) was applied at a rate of 3.5 g/kg of soil, based on standard recommendations for sodic/saline soil remediation. The amendments were thoroughly mixed into the top 15 cm of the potting substrate one week before sowing to allow for equilibration.

Biochar and agriculture gypsum have been used for soil enhancements and stabilizing soil contaminants such as soluble heavy elements or organic molecules [24]. Refs. [25,26] showed that biochar had improved plants’ performance under salt stress with an acceptable marketable value. According to [27], biochar has a potential benefit of improving soil fertility, soil properties like pH and water holding, and reducing the effects of heavy metals. On the other hand, the use of gypsum in the soil leads to decreased salinity as well as sodicity and a reduction in EC and pH [28].

Plant and soil samples were collected over three harvest stages, with soil sampled at two depths (0–15 cm and 15–30 cm) to assess vertical boron distribution. For total boron quantification, precisely 0.5 g of ground plant tissue was digested using a microwave-assisted acid digestion system following the standard EPA Method 3051 protocol for trace elements. Sample preparation and elemental analysis were performed using standardized acid digestion followed by inductively coupled plasma optical emission spectrometry an inductively (ICP-OES) by PerkinElmer (Waltham, MA, USA) (Model: 8000) (ICP-OES), consistent with established procedures, with a specific focus on boron quantification.

### 4.3. Plant Material and Growth Conditions

Four forage species commonly used in Oman’s livestock sector were selected: *Panicum maximum* (Guinea grass), *Panicum*, *Cenchrus ciliaris* (*Buffelgrass*), and *Medicago sativa* (*Alfalfa*). Seeds were sown at a rate of 10 seeds per pot and thinned to 5 uniform seedlings per pot after germination. The pots were placed in an open field under ambient temperature conditions ranging from 27 °C to 45 °C. Pots were arranged with 50 cm spacing to avoid canopy interference and re-randomized weekly to minimize microenvironmental bias.

### 4.4. Irrigation Management

Irrigation commenced 15 days after germination. Plants were irrigated to field capacity based on gravimetric measurements to prevent waterlogging and leaching. The volume of applied water was recorded at each irrigation event to precisely calculate the cumulative boron loading added to each pot over the three harvest stages.

### 4.5. Sampling and Analytical Procedures

Biomass harvesting was conducted in three stages to capture the temporal dynamics of boron uptake 30, 60, and 90 days after planting. At each harvest, plants were carefully uprooted. Shoots were severed at the soil surface, and roots were gently washed with deionized water to remove adhering soil particles at the final stage. Plant tissues were oven-dried at 65 °C to a constant weight. The dry mass of each sample was recorded to calculate specific boron concentrations. Subsequently, the dried samples were ground using a stainless-steel mill to pass through a 0.5 mm sieve for boron concentration analysis.

Soil samples were collected concurrently at two depths (0–15 cm and 15–30 cm) using a soil corer. Soil boron was extracted using the saturated paste extract method [22], which represents the standard soil–water relation for salinity and available nutrient assessments in arid soils.

### 4.6. Calculation of Bioaccumulation and Translocation Factors

To quantify internal boron partitioning and assess species-specific tolerance strategies, two widely used indices were calculated: the bioaccumulation factor (BAF = B_root_/B_soil_) and the translocation factor (TF = B_shoot_/B_root_). Where B_root_, B_shoot_, and B_soil_ represent boron concentrations (mg kg^−1^) in root tissue, aboveground shoot tissue, and the topsoil layer (0–15 cm), respectively.

### 4.7. Statistical Analysis

The experimental layout followed a completely randomized design with four replicates (*n* = 4) per treatment combination. All experimental data were analyzed using analysis of variance (ANOVA) to evaluate the main and interaction effects of irrigation water type, soil type, amendments, plant species, and harvest stage on boron accumulation. Prior to ANOVA, the normality of residuals was verified using the Shapiro–Wilk test, and homogeneity of variances was confirmed using Levene’s test. Data are presented in the figures as means ± standard deviation (SD) error bars. Mean comparisons were performed using Tukey’s Honestly Significant Difference (HSD) test at a 95% confidence level (*p* ≤ 0.05) to identify significant differences among treatments, indicated by different lowercase letters. Data processing and statistical analyses were conducted using Minitab 19.

**Figure 10:**
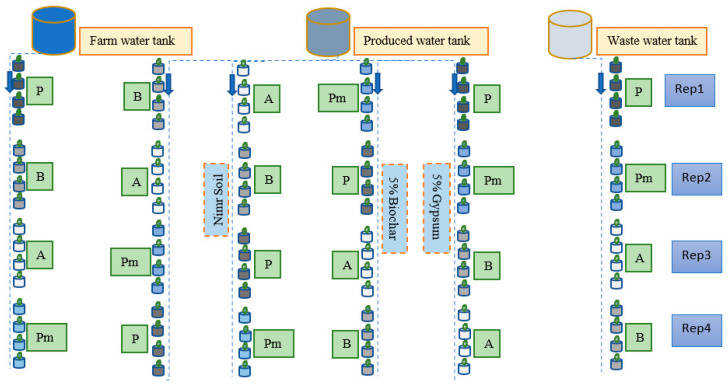
Experimental layout showing the four forage species treatments: A = *Alfalfa*, B = *Buffelgrass*, P = *Panicum*, and Pm = *Panicum maximum*. Pots contained control nutrient soil, 5% biochar, or 5% gypsum, arranged across four replicates (Rep 1–Rep 4).

**Figure 11:**
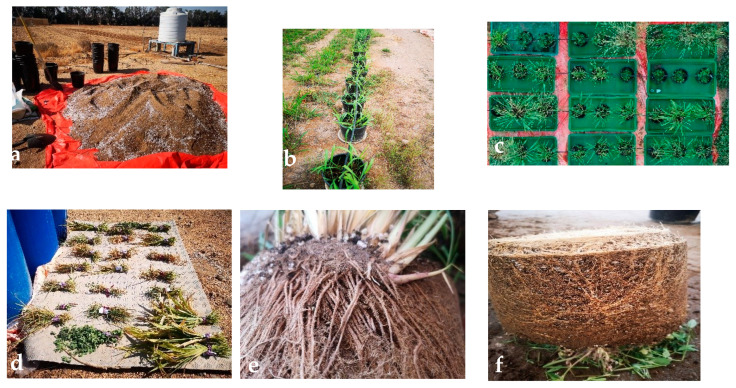

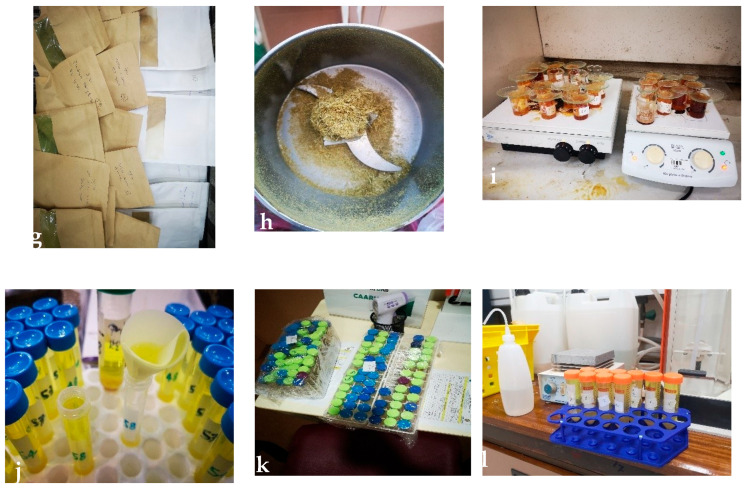
Experimental preparation and setup showing (**a**) AES soil preparation, (**b**) planting stage, (**c**) plant growth top view, (**d**) harvesting of AES plant leaves, (**e**) *Buffelgrass* roots, (**f**) pot samples showing root and soil growth, (**g**) sample labeling, (**h**) grinding of plant leaves, (**i**) sample preparation for heating, (**j**) preparation of liquid samples, (**k**) preparation of 15 mL samples, and (**l**) preparation of 50 mL samples.

Figure 10 shows a schematic diagram of the experimental layout and drip irrigation setup. The system utilized three distinct water sources: farm groundwater (FW), treated oilfield-produced water (PW), and treated wastewater (WW). The experimental plots contained four forage species, represented in the layout by the following abbreviations: A = *Alfalfa* (*Medicago sativa*); b = *Buffelgrass* (*Cenchrus ciliaris*); p = *Panicum* (*Panicum antidotale*); and Pm = *Panicum maximum*. The plants were grown in three different soil/amendment substrates: nutrient soil (control), soil amended with 5% biochar, and soil amended with 5% gypsum. The experiment was arranged with four independent replicates (Rep 1–Rep 4) for each treatment combination (Figure 11).

Studies of [29,30,31] outlined the constraints imposed by various mineral elements on plants and soil. The limitations on boron (B) are highlighted as critical, with 100 mg kg^−1^ in the soil and between 283 and 333 mg kg^−1^ in the plants as the feeding limit.

## 5. Conclusions

This study demonstrates that boron accumulation represents a critical limitation for the use of treated oilfield-produced water (PW) in forage production, but species-specific management can mitigate these risks. By calculating bioaccumulation factors (BAFs) and translocation factors (TFs), our data indicates that forage grasses employ divergent internal boron-handling strategies under high-salinity stress. *Alfalfa* exhibited a strong tendency to translocate boron to aerial tissues (frequently yielding TF values greater than 1), suggesting a high risk of excessive boron accumulation in the harvestable shoot biomass. In contrast, *Buffelgrass* demonstrated a restrictive behavior (consistently maintaining TF values well below 1), indicating an effective root-level exclusion mechanism which may reduce boron accumulation in aboveground tissues.

From an applied perspective, the safe reuse of treated oilfield-produced water requires moving beyond standard salinity indicators to focus on ion-specific dynamics. Furthermore, while nature-based solutions are frequently proposed for contaminant remediation, their application must be context-specific. In this study, biochar amendments were paradoxically associated with elevated root bioaccumulation (increased BAF), acting as a boron availability enhancer rather than an immobilizer under extreme salinity, likely due to complex ion competition. Therefore, relying solely on biochar or gypsum as a standalone mitigation strategy for boron in PW reuse systems requires further validation under varying data conditions.

While this study provides valuable insights into boron transport patterns, we acknowledge that definitive conclusions regarding acute phytotoxicity and actual yield losses require long-term biomass and physiological data, which were beyond the scope of this targeted ion-uptake study. Nevertheless, the observed partitioning trends provide a robust foundation for agricultural management. Prioritizing low-translocation species like *Buffelgrass*, alongside routine monitoring of soil-profile boron and shoot tissue composition, represents a practical and evidence-based management approach for the sustainable and safe utilization of alternative water resources in arid regions.

## Figures and Tables

**Table 1:** Boron mineral concentration of the soils used in the experiment.

Minerals (mg/kg)	Nimr Soil	Control Soil
B	0.03	0.013

**Table 2:** Boron mineral concentration of the oilfield-produced water, treated wastewater, and groundwater used in the SQU-AES experiment one.

Water-Minerals Concentration (mg L^−1^)	Oilfield-Produced Water	Treated Wastewater	Farm Groundwater
B	6.95	1.17	0.23

**Table 3:** Summary of third-party water analysis data for two samples collected upstream and downstream of the treatment unit [18].

Parameter Inflow	Inflow (mg L^−1^)	Final Outflow (mg L^−1^)
Total dissolved solids	6500–7000	12,000
Suspended solids	28	10
Oil in water	280	<0.5
BOD	15.7	<1
Total nitrogen	2.5	<0.5
Total phosphorus	0.03	<0.5
Boron	4.5	5.6

## Data Availability

The original contributions presented in this study are included in the article. Further inquiries can be directed to the corresponding author.

## Abbreviations

The following abbreviations are used in this manuscript:

Alfa	*Alfalfa* (Medicago sativa)
b	*Buffelgrass* (Cenchrus ciliaris)
EC	Electrical Conductivity
FW	Farm groundwater
p	*Panicum* (Guinea grass)
Pmax	*Panicum maximum* (Guinea grass)
PW	Oilfield-produced water
R/RO	Reverse Osmosis
TDS	Total dissolved solids
W/Well	Ground well water
